# The prognostic value of ^18^F-PSMA-1007 PET/CT in predicting pathological upgrading of newly diagnosed prostate cancer from systematic biopsy to radical prostatectomy

**DOI:** 10.3389/fonc.2023.1169189

**Published:** 2023-05-10

**Authors:** Anqi Zheng, Zhuonan Wang, Liang Luo, Ruxi Chang, Jungang Gao, Bo Wang, Xiaoyi Duan

**Affiliations:** PET/CT Center, The First Affiliated Hospital of Xi’an Jiaotong University, Xi’an, China

**Keywords:** prostate cancer, biopsy, radical prostatectomy, Gleason grade group, ^18^F-PSMA-1007 PET/CT

## Abstract

**Objective:**

This study aimed to evaluate predictors for upgrading of newly diagnosed prostate cancer from systematic biopsy (SB) to radical prostatectomy (RP) using fluorine-18 prostate-specific membrane antigen 1007 (^18^F-PSMA-1007) positron emission tomography/computed tomography (PET/CT) and association with clinical parameters.

**Materials and methods:**

We retrospectively collected data from biopsy-confirmed prostate cancer (PCa) patients who underwent ^18^F-PSMA-1007 PET/CT prior to RP from July 2019 and October 2022. Imaging characteristics derived from ^18^F-PSMA-1007 PET/CT and clinical parameters were compared in patients of pathological upgrading and concordance subgroups. Univariable and multivariable logistic regressions were performed to analyze factors predicting histopathological upgrading from SB to RP specimens. Discrimination ability of independent predictors was further evaluated by receiver operating characteristic (ROC) analysis with corresponding area under the curve (AUC).

**Results:**

Pathological upgrading occurred in 26.97% (41/152) PCa patients, and 23.03% (35/152) of all patients experienced pathological downgrading. Concordance rate reached 50% (76/152). International Society of Urological Pathology grade group (ISUP GG) 1(77.78%) and ISUP GG 2 (65.22%) biopsies were related with the highest rate of upgrading. Multivariable logistic regression analyses showed that prostate volume (OR= 0.933; 95% CI, 0.887–0.982; p = 0.008), ISUP GG 1 *vs*. 4 (OR= 13.856; 95% CI: 2.467–77.831; p = 0.003), and total uptake of PSMA-avid lesions (PSMA-TL) (OR = 1.003; 95% CI, 1.000–1.006; p = 0.029) were found to be independent risk factors of pathological upgrading after RP. The AUCs and corresponding sensitivity and specificity of the independent predictors of synthesis for upgrading were 0.839, 78.00%, and 83.30% respectively, which showed good discrimination capacity.

**Conclusion:**

^18^F-PSMA-1007 PET/CT may help to predict pathological upgrading between biopsy and RP specimens, particularly for ISUP GG 1 and ISUP GG 2 patients with higher PSMA-TL and smaller prostate volume.

## Introduction

The developing clinical management of prostate cancer (PCa), where the Gleason Score (GS) is still regarded as a crucial determinant for the choice of treatment, has been significantly impacted by International Society of Urological Pathology (ISUP) grade at biopsy (1). In recent years, the difference in ISUP grade from systematic biopsy (SB) to radical prostatectomy (RP) remains an important issue faced in clinical practice, with upgrading common after RP (2–4). Upgrading from biopsy to RP results in relative undertreatment (5). Increased prostatectomy grade group (GG) is connected to a lower prostatic cancer-specific survival rate and a higher postoperative biochemical recurrence rate (4). Moreover, in patients who do not undergo surgery, biopsy GS remains the key factor for treatment decisions and prognostication (6).

^18^F-Labeled prostate-specific membrane antigen 1007 (^18^F-PSMA-1007) has emerged as one of the most sensitive primary staging tools for PCa (7, 8). PSMA is a type II transmembrane glycoprotein that is highly overexpressed in PCa tumors and metastasis and is associated with PCa progression and prognosis (9, 10). However, heterogeneity in PSMA expression and pathological findings at biopsy might contribute to therapy selection bias, which may influence surgical decision particularly. Patients with low-grade prostate cancer who have advancing pathology can require more extensive surgery or possibly judge not to have surgery (11, 12). There is limited research on whether ^18^F-PSMA-1007 positron emission tomography/computed tomography (PET/CT) can predict pathological upgrading from biopsy to RP.

The objective of this study was to determine whether ^18^F-PSMA-1007 PET/CT might be used as a biomarker to predict the pathological upgrading from SB to RP. By combination of ^18^F-PSMA-1007 PET/CT derived from parameters with clinical variables, we expect to improve the predictive accuracy of ^18^F-PSMA-1007 PET/CT in predicting SB to RP-grade concordance.

## Materials and methods

### Patients

**Figure 1:**
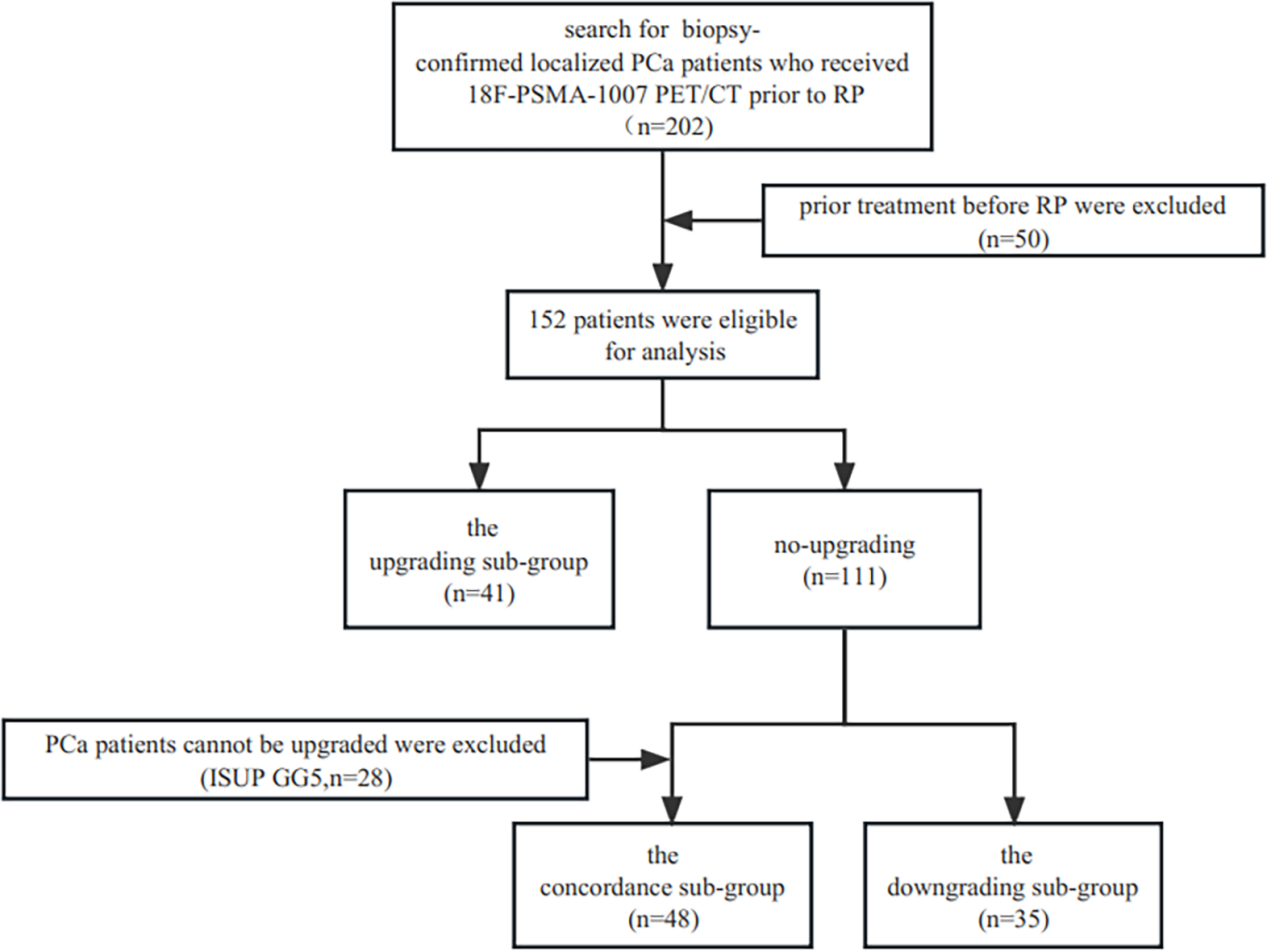
Flowchart of the study.

The study has been approved by the institutional review board (No. 2019LSYZD-J1-H) in compliance with the Declaration of Helsinki, and all subjects signed an informed consent form. A total of 202 consecutive biopsy-confirmed localized adenocarcinoma of prostate patients between July 2019 and October 2022 who received ^18^F-PSMA-1007 PET/CT prior to RP were included retrospectively. Patients who underwent radiation therapy, androgen deprivation therapy, or both and those with insufficient pathological or clinical data were not included in the study. Finally, 152 patients were eligible for analysis. Upgrading and downgrading were defined as increase or decrease from biopsy ISUP GG group to another ISUP GG group in RP, respectively. The ISUP GG groups were ISUP GG 1 (3 + 3 = 6), ISUP GG 2 (3 + 4 = 7), ISUP GG 3 (4 + 3 = 7), ISUP GG 4 (4 + 4 = 8, 3 + 5 = 8, 5 + 3 = 8), and ISUP GG 5 (4 + 5 = 9, 5 + 4 = 9, 5 + 5 = 10). In this study, we separated the cohort into three sub-groups: the upgrading sub-group, the concordance sub-group, and the downgrading sub-group. The ISUP GG 5 (n=28) at biopsy was not included in the comparison of the upgrading and concordance groups because upgrading could not take place there. The flowchart of patient enrollment is provided in **Figure 1**.

### ^18^F-PSMA-1007 and image acquisition

PET/CT images were obtained on a PET/CT scanner (Gemini 64TF, Philips, Netherlands). A completely automated radiopharmaceutical synthesis equipment based on a modular architecture was used for radiolabeling (MINItrace, GE Healthcare, USA). The radiochemical purification yield of ^18^F-PSMA-1007 was over 99% and was tested using both radiothin layer chromatography and high-performance liquid chromatography. Patients underwent a 90-min PET/CT scan after receiving an intravenous injection of ^18^F-PSMA-1007 PET/CT (3.7 MBq/kg body weight). Low-dose CT scans were acquired from the head to the proximal thighs for PET attenuation (pitch, 0.8 mm; automatic mA, 140 kV [peak]; tube single turn rotation time, 1.0 s; and 5-mm slice thickness). As in our prior work (13), whole-body PET scans were conducted in three dimensions (emission time of 90 s per bed position, scanned at a total of 7–10 beds).

### Imaging analysis

All ^18^F-PSMA-1007 PET/CT imaging was independently evaluated by two board-certified nuclear medicine physicians with over 10 years of PET/CT experience, who were blind to the pathological outcomes of biopsy and final RP specimens. When the views of the two specialists differed, consensus was reached through debate. Focal uptake of ^18^F-PSMA-1007 in prostatic regions higher than the background was reported as suspicious for malignancy (14). All ^18^F-PSMA-1007 PET/CT images were analyzed using Fusion Viewer software in the Extended Brilliance Workstation (EBW, Philips, Netherlands). Volumes of interest (VOIs) were delineated on continuous PET scans for each lesion. The maximum standardized uptake values (SUVmax), the mean standardized uptake values (SUVmean), total volume of PSMA-avid tumor (PSMA-TV), and total uptake of PSMA-avid lesions (PSMA-TL) of the primary PCa were obtained by drawing automatically with a manually adapted VOI with a 40% isocontour threshold of the SUVmax centered on lesions with focally increased uptake corresponding to the tumor site. If necessary, adjustment on the axial and sagittal planes was made. PSMA-TL was calculated by multiplying the PSMA-TV and SUVmean.

### Histopathological analysis

All patients underwent a 12–14 core transrectal ultrasound (TURS)-guided systematic prostate biopsy and RP. All RP specimens were serially sectioned and sent to the pathology department in their entirety for histological evaluation. Two urological pathologists with more than 10 years of experience independently performed RP histopathological specimens using the 2014 ISUP Gleason Grading Guidelines. The overall biopsy GS was obtained using the highest GS core. The overall GS in RP specimens with multifocal disease was calculated using the nodule with the greatest GS.

### Statistical analysis

For continuous variables not fitting into a normal distribution, the median and interquartile range (IQR) were used; meanwhile, for categorical variables, numbers and proportions were used. Continuous variables were compared using analysis of *t*-test or Wilcoxon–Mann–Whitney U-test and categorical variables were compared using χ^2^-test. Univariate and multivariable logistic regression analyses were conducted to evaluate the factors related with biopsy to RP upgrading and downgrading. To arrive at the final model, those that were not significant at p < 0.05 were eliminated one at a time from the whole model, analyzing the stability of the regression coefficients to assess potential confounding. Area under the curve (AUC) calculated from receiver operating characteristic (ROC) was used to evaluate the independent predictors’ capacity for discrimination. Statistical analyses were performed using IBM SPSS Statistics version 26.0 and MedCalc version 20.022. p < 0.05 was used to determine statistical significance for all tests.

## Results

A total of 152 patients were finally included in our study. The clinical and imaging characteristics of the patients who were included are summarized in **Table 1**. Pretreatment PSA levels ranged between 1.66 and 367.2 ng/ml. Most patients presented with a PSA level ≥ 10 ng/ml (82.24%) and 2 (92.4%) had PSA values under 4 ng/ml. In 108 (71.05%), the prostate volume was under 40 ml. The interval between the ^18^F-PSMA-1007 PET/CT scan, prostate systematic biopsy, and RP was less than a month.

**Table 1:** Clinical and imaging characteristics of patients who were included.

Patients(n)	152
Age, mean ± SD (range)	69.32 ± 6.98 (49–86)
PSA level, ng/ml	18.48 (11.57–33.57)
PSA density, ng/m^2^	0.53 (0.34–1.11)
Prostate vulume, ml	32 (25.25–44.00)
SUVmax,median (IQR)	16.78 (9.58–22.44)
SUVmean,median (IQR)	6.63 (4.80–8.35)
PSMA-TV, cm^3^	18.70 (8.95–27.42)
PSMA-TL,median (IQR)	119.50 (44.28–196.74)
No. of positive cores, median (IQR)	4 (2–8)
Percentage of positive cores at SB (%)	46.86 (25.00–66.67)
SVI on PET/CT (%)	
Positive	31 (20.39)
Negative	121 (79.61)
Tumor family history (%)	
Positive	33 (21.71)
Negative	119 (78.29)
Clinical T stage (%)	
cT1	3 (1.97)
cT2	122 (80.26)
cT3a	3 (1.97)
cT3b	22 (14.47)
cT4	2 (1.32)
GS at SB (%)	
6	18 (11.84)
7	52 (34.21)
8	43 (28.29)
9	34 (22.37)
10	5 (3.29)
ISUP GG at SB (%)	
1	18 (11.84)
2	23 (15.13)
3	29 (19.08)
4	43 (28.29)
5	39 (25.66)

PSA, prostate specific antigen; SUVmax, the maximum standardized uptake values; SUVmean, the mean standardized uptake values; PSMA-TV, total volume of PSMA-avid tumor; PSMA-TL, total uptake of PSMA-avid lesions; SVI, seminal vesicle invasion; GS, Gleason grade; ISUP, International Society of Urological Pathology; GG, grade group; SB, systematic biopsy.

The distribution between the biopsy GG and the final RP GG, and the corresponding upgrades, downgrades, and concordance are shown in **Table 2**. After histopathological examination, GG was not changed in 76 (50.00%) of the patients, but it was upgraded in 41 (26.97%) and downgraded in 35 (23.03%) of the patients at RP. ISUP GG 1 (77.78%) and GG 2 (65.22%) biopsies were related with the highest rate of upgrading. The total incidence of biopsy GG 1 upgrading was 14 (77.78%) of 18 patients, with the majority (50.00%, n = 7) upgrading to GG 3, followed by GG 2 (35.71%, n = 5) and GG 4 (14.29%, n = 2). The highest concordance was found in biopsy GG 3 (75.86%) and biopsy GG 5 (71.79%). More than half (51.16%) of the biopsy GG 4 cases were downgraded, the vast majority (39.53%) to GG 3, and this was the most prevalent biopsy-to-RP downgrading.

**Table 2:** Global grade groups on biopsy and radical prostatectomy and grade change (n = 152).

GS/GG at biopsy	GS/GG at RP [N (% of GS/GG)]						Change in score [N (% of GS/GG)]		
	6 (GG 1)	3 + 4 = 7 (GG 2)	4 + 3 = 7 (GG 3)	8 (GG 4)	9-10 (GG 5)	total	Upgrade	concordance	downgrade
6 (GG 1)	4 (22.22)	5 (27.78)	7 (38.89)	2 (11.11)	0	18	14 (77.78)	4 (22.22)	0
3 + 4 = 7 (GG 2)	0	8 (34.78)	9 (39.13)	4 (17.39)	2 (8.70)	23	15 (65.22)	8 (34.78)	0
4 + 3 = 7 (GG 3)	2 (6.90)	0	22 (75.86)	4 (13.79)	1 (3.45)	29	5 (17.24)	22 (75.86)	2 (6.90)
8 (GG 4)	1 (2.33)	4 (9.30)	17 (39.53)	14 (32.56)	7 (16.28)	43	7 (16.28)	14 (32.56)	22 (51.16)
9–10 (GG 5)	1 (2.56)	2 (5.13)	4 (10.26)	4 (10.26)	28 (71.79)	39	0	28 (71.79)	11 (28.21)

GS, Gleason grade; GG, grade group; RP, radical prostatectomy.

Compared to the concordance group, patients in the upgrading group had higher SUVmax, lower GS at biopsy, and lower ISUP GG at biopsy (p = 0.044, p = 0.009, p < 0.001, respectively) (**Table 3**). Baseline characteristics of prostate-specific antigen (PSA), PSA density, prostate volume, SUVmean, PSMA-TV, PSMA-TL, and seminal vesicle invasion (SVI) on PET/CT in the upgrading group and concordance group did not substantially differ.

**Table 3:** Demographics of patients with pathological upgrading and concordance subgroups.

	Pathological upgrading status		
Characteristics	Upgrading (n=41)	Concordance (n=48)	P
Age, mean ± SD (range)	66.95 ± 6.16	69.06 ± 6.51	0.122
PSA level, ng/ml	17.68 (11.30–29.65)	21.06 (11.92–34.88)	0.466
PSA density, ng/m^2^	0.71 (0.39–1.32)	0.74 (0.35–1.17)	0.742
Prostate vulume, ml	29.00 (23.50–32.00)	33.00 (24.25–43.75)	0.052
SUVmax, median (IQR)	19.56 (14.88–28.84)	15.58 (8.98–26.10)	0.044
SUVmean, median (IQR)	6.77 (5.31–8.27)	6.75 (4.98–8.30)	0.489
PSMA-TV, cm^3^	20.80 (12.06–31.19)	19.04 (12.82–26.76)	0.537
PSMA-TL, median (IQR)	142.40 (71.78–226.40)	105.65 (57.27–184.15)	0.340
**SVI on PET/CT (%)**			0.527
Positive	9 (21.95)	8 (16.67)	
Negative	32 (78.05)	40 (83.33)	
**Tumor family history (%)**			0.670
Positive	11 (26.83)	11 (22.92)	
Negative	30 (73.17)	37 (77.08)	
**GS at SB (%)**			0.009
6	14 (34.15)	4 (8.33)	
7	20 (48.78)	30 (62.50)	
8	7 (17.07)	14 (29.17)	
9	0 (0)	0 (0)	
10	0 (0)	0 (0)	
**ISUP GG at SB (%)**			<0.001
1	14 (34.15)	4 (8.33)	
2	15 (36.59)	8 (16.67)	
3	5 (12.20)	22 (45.83)	
4	7 (17.07)	14 (29.17)	
5	0 (0)	0 (0)	

PSA, prostate specific antigen; SUVmax, the maximum standardized uptake values; SUVmean, the mean standardized uptake values; PSMA-TV, total volume of PSMA-avid tumor; PSMA-TL, total uptake of PSMA-avid lesions; SVI, seminal vesicle invasion; GS, Gleason grade; ISUP, International Society of Urological Pathology; GG, grade group; SB, systematic biopsy.

Univariable and multivariable logistic regression analyses of clinical, pathological, and imaging parameters for the prediction of pathology upgrading are shown in **Table 4**. Of note, upgraded patients had a higher PSMA-TL, smaller prostate volume, and lower ISUP GG at biopsy. Univariable analyses demonstrated that prostate volume, biopsy ISUP GG, and biopsy GS were significantly associated with pathological upgrading from biopsy to final RP specimens. In multivariable logistic regression analyses, only prostate volume (OR= 0.933; 95% CI, 0.887–0.982; p = 0.008), ISUP GG 1 *vs*. 4 (OR= 13.856; 95% CI, 2.467–77.831; p = 0.003), and PSMA-TL (OR = 1.003; 95% CI, 1.000–1.006; p = 0.029) were found to be independent risk factors of pathological upgrading after RP.

**Table 4:** Univariable and multivariable logistic regression analyses of possible predictors for pathological upgrading from SB to RP.

	Univariable logistic regression			Multivariable logistic regression		
Parameters	OR	95%CI	P	OR	95%CI	p
Age	0.948	0.886–1.015	0.124			
PSA level, ng/ml	1.005	0.994–1.016	0.381			
PSA density, ng/m^2^	1.333	0.840–2.114	0.223			
Prostate vulume, ml	0.947	0.907–0.988	0.012	0.933	0.887–0.982	0.008
SUVmax	1.024	0.993–1.055	0.128			
SUVmean	1.058	0.904–1.239	0.483			
PSMA-TV, cm^3^	1.014	0.986–1.043	0.340			
PSMA-TL	1.001	0.999–1.004	0.311	1.003	1.000–1.006	0.029
SVI on PET/CT	1.406	0.487–4.058	0.528			
Tumor family history	1.233	0.470–3.236	0.670			
cT(1–5)	1.440	0.815–2.545	0.209			
No. of positive cores	0.893	0.781–1.021	0.097			
Percentage of positive cores	0.252	0.048–1.315	0.102			
GS at SB						
6 *vs*. 8	7.000	1.668–29.384	0.008			
7 *vs*. 8	1.333	0.458–3.884	0.598			
ISUP GG at SB						
1 *vs*. 4	7.000	1.668–29.384	0.008	13.856	2.467–77.831	0.003
2 *vs*. 4	3.750	1.076–13.073	0.038	3.699	0.951–14.384	0.059
3 *vs*. 4	0.455	0.120–1.717	0.245	0.362	0.085–1.546	0.170

PSA, prostate specific antigen; SUVmax, the maximum standardized uptake values; SUVmean, the mean standardized uptake values; PSMA-TV, total volume of PSMA-avid tumor; PSMA-TL, total uptake of PSMA-avid lesions; SVI, seminal vesicle invasion; cT (1–5; T1 = 1, T2 = 2, T3a=3, T3b=4, T4 = 5); GS, Gleason grade; ISUP, International Society of Urological Pathology; GG, grade group; SB, systematic biopsy.

**Figure 2:**
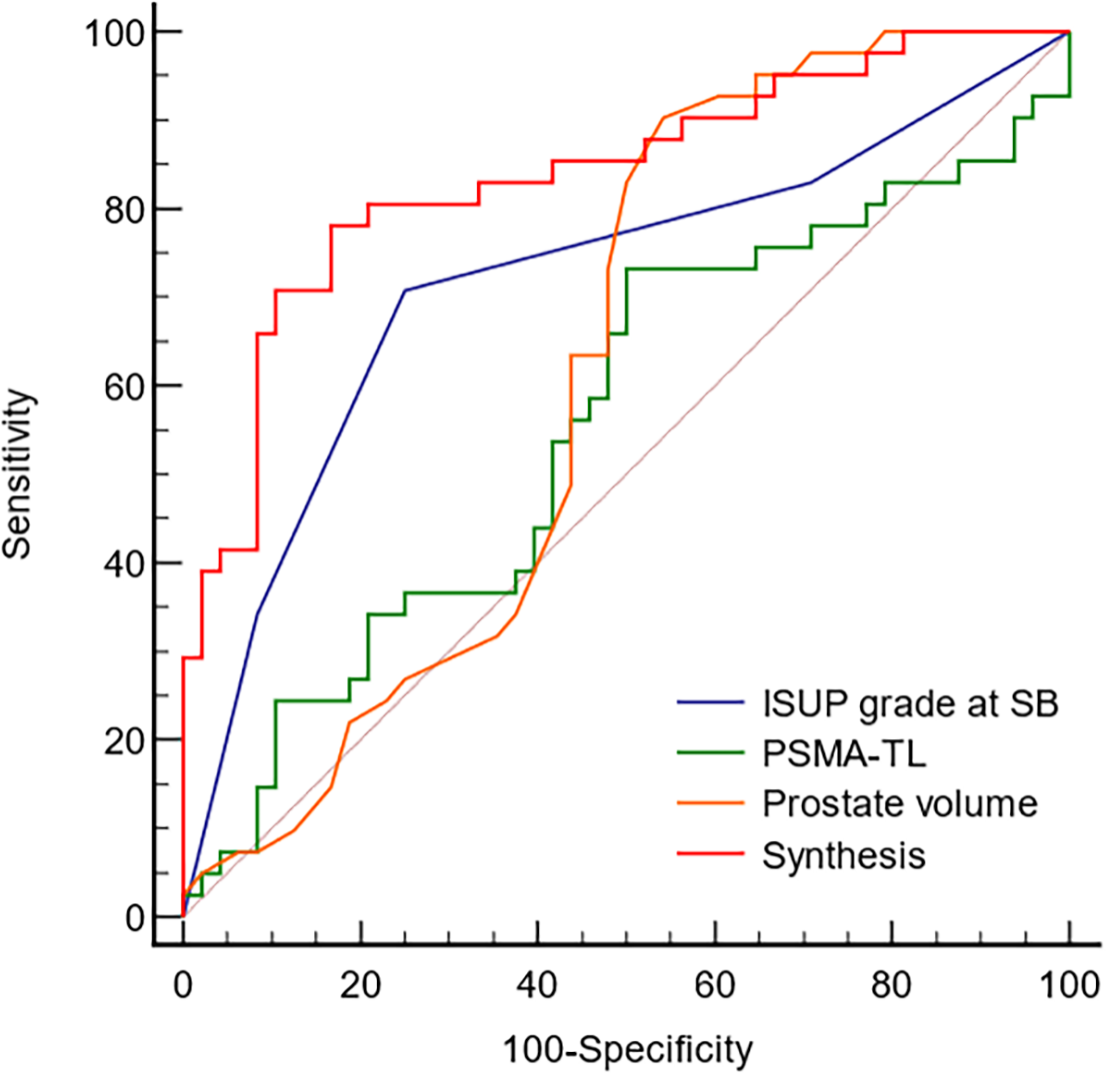
The ROC curve analyzed the diagnostic accuracy of the parameters determined by upgrades. ISUP, International Society of Urological Pathology; PSMA-TL, total uptake of PSMA-avid lesions; Synthesis, ISUP grade at SB, PSMA-TL and prostate volume.

The ROC curve (**Figure 2**) was used to examine the diagnostic accuracy of the parameters for upgrading determined by the previous multivariate logistic regression model. The AUC and corresponding sensitivity, specificity, and cutoff values for each predictor of upgrading and downgrading are displayed in **Supplementary Table S1**. In comparison to other independent risk indicators, the synthesis of PSMA-TL, prostate volume, and ISUP grade at SB demonstrated good discriminate ability in predicting pathological upgrading with an AUC of 0.839 (95% CI, 0.746–0.908). The ROC calculated the cutoff values for each predictor, and their synthesis for upgrading from SB to RP was ISUP grade at SB ≤ 2, PSMA-TL ≥ 95.268, prostate volume ≤ 34 ml, and the synthesis ≥ 0.453.

## Discussion

The clinical stage, PSA level, and biopsy GG are the major factors in determining PCa risk stratification (15, 16). Prior studies show that upgrading from biopsy to RP specimens frequently takes place in the clinic, which leads to the inaccurate assignment of risk classification (3, 15, 17). It is urgent to improve the accuracy of predicting risk stratification. PSMA PET/CT is increasingly being utilized for PCa primary staging (18, 19). ^68^Ga-PSMA-11 PET/CT was proven to be an appropriate biomarker for the prediction of pathological upgrading in a recent investigation (20). However, the study did not further examine tumor burden determined by PSMA PET/CT, including SUVmean, PSMA-TV, and PSMA-TL. ^18^F-PSMA-1007 is primarily excreted by the biliary tract and has advantages of detecting lesions in the periphery of localized prostate cancer (21). However, few studies are done on the use of ^18^F-PSMA-1007 PET/CT as a risk factor to predict pathological upgrading from biopsy to final RP specimens. Therefore, this study was designed to investigate whether ^18^F-PSMA-1007 PET/CT can be used as a biomarker to predict pathological upgrading from SB to ultimate RP.

Our study found that biopsies GG 1 and GG 2 patients had the highest risk of updating, with the proportion of upgrading being 77.78% (14/18) and 65.22% (15/23), respectively. ISUP GG 1 patients were primarily upgraded to ISUP GG 2 and ISUP GG 3, and ISUP GG 2 were largely upgraded to GG 3. Active surveillance (AS) might not be a good option for these ISUP GG ≤ 2 individuals because it would raise their risk of tumor progression and mortality (22, 23). Similarly, Altok et al. observed that 70.9% of biopsy GG 1 patients in their study cohort were upgraded at RP, with the majority of them being upgraded to GG 2 (17). Patients with biopsy GG 2 had a 44.6% upgrade rate, according to Pham et al. (24). The insufficiency of our sample size might be the cause of this discrepancy in proportions.

Lower biopsy GG, higher PSMA-TL, and smaller prostate volume were related with a significantly increased probability of ISUP GG upgrading, according to the current study. ISUP GG, particularly ISUP GG 1 *vs*. 4, was found to be an independent predictor of pathological upgrading. A focus of prostate cancer with a higher GG could be missed by a TURS-guided systematic prostate biopsy, since it only represents a small, randomly selected portion of the total prostatic tissue (11, 25). Inter- and interobserver variability in biopsy Gleason assignment may be one of the reasons for pathological upgrading. PSMA-TL was another independent predictor of pathological upgrading. Routine parameters from ^18^F-PSMA-1007 PET/CT, such as SUVmax, SUVmean, and SUVmin, are not adequate to capture the full extent of the disease and the tumor burden. PSMA-TL, a quantitative assessment of tumor load, was shown to correlate significantly with GS and PSA in a previous study (26). PSMA-TL is increasingly used to assess treatment response to predict prognosis and guide treatment (26–28). Prostate volume ≤34 ml was also an independent predictor of pathological upgrading in our study according to the multivariate logistic regression analysis. Xu et al. showed that PV < 30 ml (p<0.001) was an independent predictor of upgraded GS (29). Biopsy GG, prostate volume, and patient year were confirmed to be individual predictors of GG upgrading in the study of Yan et al. (30). The results of both investigations supported our findings, demonstrating the importance of smaller prostate volume in predicting pathological upgrading at RP. Smaller prostates have higher pathological grades and exhibit biologically more aggressive behavior in a previous study, which could explain our findings (29).

Additionally, we observed that a 23.03% rate of downgrading and biopsy GG 4 (51.16%) had the greatest risk for pathological downgrading. This finding suggests that some patients with biopsy GG 4 are frequently overstated. These patients in our study were consequently downgraded from NCCN high-risk PCa to low-intermediate risk. Pathological analysis of the prostatectomy specimens may miss ISUP GG 4 due to the diagnosis of its small lesions (31). The rate of downgrading may be impacted by inadequate gland sampling on final RP pathological specimens (32).

There are certain limitations to our study. To begin with, this is a single-center retrospective study with a limited sample size, which could lead to our results not being widely applicable. A large prospective multicenter study is needed for further validation. Second, there may have been more cases of upgrades and downgrades in our study because all the patients included underwent only systematic rather than targeted biopsies (TB). Studies have reported that MRI-targeted prostate biopsy leads to less pathologic upgrading and equal downgrading in comparison to systematic biopsy. Third, only patients with biopsy-proven prostate cancer who underwent RP were included in this analysis, resulting in a selection bias that excluded patients who did not undergo RP surgery. Last but not least, only tPSA and PSA density were included in our analysis, which also has the limitation of not adding extra prostate cancer markers. There are many prostate cancer markers, but they mainly include PSA, PSA-related derivatives, and genetic markers (2, 3). However, PSA is still the most widely used in clinical practice. tPSA did not predict pathological upgrading after RP in our research, in line with the conclusions of Yin et al. in a study using ^68^Ga-PSMA-11 PET/CT (20). Similarly, another study simultaneously included four PCa markers, namely, tPSA, fPSA, fPSA/tPSA, and PSA density (22). Except PSA density, the other three were not statistically significant in the multivariate logistic regression analysis. However, it has also been demonstrated that tPSA and PSA densities are independent risk factors for pathological upgrading after RP in PCa with GS 3 + 4 (24). This may be related to the selection time of PSA screening, PSA range, treatment at the same time, different treatment methods, and different prostate volume measurement methods. There are few studies of genetic markers in predicting PCa upgrading. Wang et al. found that miR-145-5p was an independent predictor of upgrading in patients with GS 3 + 3 PCa at the molecular and genetic levels (33). PSA testing in conjunction with RARB, RASSF1, and GSTP1 DNA methylation can be employed as a biomarker for PCa upgrading (34). However, there is one advantage to our research. PSMA is not expressed or low expressed in approximately 5%–10% of prostate cancer patients; prior studies illustrated these portions of patients mainly seen in severely treated patients receiving multiple chemotherapy and androgen deprivation therapy, poorly differentiated tumors with neuroendocrine differentiation, and dedifferentiated adipocytic carcinoma (35, 36). None of the patients included in this study had received any antineoplastic therapy at the time of the PET/CT examination, and all had prostate adenocarcinoma. As a result, this may improve the predictive power of PSMA PET/CT appropriately.

In conclusion, ^18^F-PSMA-1007 PET/CT in conjunction with clinical data may be useful to evaluate individual risk and assign patients to a more precise risk stratification. Clinicians should pay closer attention to ISUP GG 1 and GG 2 patients at initial biopsy patients with higher PSMA-TL and smaller prostate volume.

## Data availability statement

The data that support the findings of this study are available from the corresponding author upon reasonable request.

## Ethics statement

The study has been approved by the institutional review board (No. 2019LSYZD-J1-H) compliance with the Declaration of Helsinki. The patients/participants provided their written informed consent to participate in this study. Written informed consent was obtained from the individual(s) for the publication of any potentially identifiable images or data included in this article.

## Author contributions

AZ drafted the manuscript and contributed to the conception and design, analysis, and interpretation of data. ZW and LL contributed to the analysis and interpretation of data. RC, JG, and BW contributed to acquisition of data. ZW and XD contributed to the revision of the manuscript critically for important intellectual content. All authors contributed to the article and approved the submitted version.
