# Supplementary material for: The prognostic value of 18F-PSMA-1007 PET/CT in predicting pathological upgrading of newly diagnosed prostate cancer from systematic biopsy to radical prostatectomy
Source: Front Oncol. 2023 May 10;13:1169189. doi: 10.3389/fonc.2023.1169189 (PMC10206242; doi:10.3389/fonc.2023.1169189)
Supplement: Supplementary file 1 [file Table_1.docx]

**Supplemental table 1**

|  | AUC (95% CI) | Cutoff | Sensitivity | specificity |
| --- | --- | --- | --- | --- |
| ISUP grade at SB | 0.721 (0.615~0.811) | 2 | 70.73 | 75.00 |
| PSMA-TL | 0.559 (0.450~0.664) | 95.268 | 73.2 | 50.00 |
| Prostate volume | 0.620 (0.511~0.721) | 34 | 90.24 | 45.83 |
| synthesis | 0.839 (0.746~0.908) | 0.453 | 78.0 | 83.3 |
